# Re-Introduction of Bovine Viral Diarrhea Virus in a Disease-Free Region: Impact on the Affected Cattle Herd and Diagnostic Implications

**DOI:** 10.3390/pathogens10030360

**Published:** 2021-03-18

**Authors:** Kerstin Albrecht, Miriam Linder, Anja Heinrich, Jennifer Höche, Martin Beer, Wolfgang Gaede, Kerstin Wernike

**Affiliations:** 1 Department of Veterinary Medicine, State Institute for Consumer Protection of Saxony-Anhalt, 39576 Stendal, Germany; miriam.linder@sachsen-anhalt.de (M.L.); anja.heinrich@sachsen-anhalt.de (A.H.); jennifer.hoeche@sachsen-anhalt.de (J.H.); wolfgang.gaede@sachsen-anhalt.de (W.G.); 2 Institute of Diagnostic Virology, Friedrich-Loeffler-Institut, 17493 Greifswald—Insel Riems, Germany; martin.beer@fli.de (M.B.); kerstin.wernike@fli.de (K.W.)

**Keywords:** bovine viral diarrhea, pestivirus, flavivirus, diagnostics, serology, eradication program, control, epidemiology

## Abstract

Bovine viral diarrhea (BVD) is one of the most important infectious cattle diseases worldwide. The major source of virus transmission is immunotolerant, persistently infected (PI) calves, which makes them the key target of control programs. In the German federal state of Saxony-Anhalt, a very low prevalence was achieved, with more than 99.8% of the cattle herds being free from PI animals since the year 2013. In 2017, BVD virus was detected in a previously disease-free holding (herd size of ~380 cows, their offspring, and fattening bulls). The purchase of two so-called Trojan cows, i.e., dams pregnant with a PI calf, was identified as the source of infection. The births of the PI animals resulted in transient infections of in-contact dams, accompanied by vertical virus transmission to their fetuses within the critical timeframe for the induction of PI calves. Forty-eight days after the birth of the first PI calf, all animals in close contact with the Trojan cows during their parturition period were blood-sampled and serologically examined by a neutralization test and several commercial ELISAs. The resulting seroprevalence strongly depended on the applied test system. The outbreak could be stopped by the immediate elimination of every newborn PI calf and vaccination, and since 2018, no BVD cases have occurred.

## 1. Introduction

Bovine viral diarrhea (BVD) is caused by a pestivirus of the family *Flaviviridae* that exists in the two species BVDV-1 (syn. *Pestivirus A*) and BVDV-2 (syn. *Pestivirus B*) [1]. Both species are further divided into subtypes. Independent of the species, BVDV isolates are classified according to their in vitro growth in cell cultures into the cytopathic (cp) and non-cytopathic (ncp) biotypes.

BVD is one of the most significant cattle diseases worldwide, inducing major direct and indirect economic losses, including losses for corresponding control programs [2,3,4,5,6]. Furthermore, BVD has a substantial impact on animal welfare in the beef and dairy industries. The clinical signs of BVD range from clinically inapparent infections or mild to moderate unspecific symptoms, such as fever, diarrhea, and pneumonia, to severe acute cases characterized by hemorrhagic syndromes and mucosal disease-like lesions [7]. Fetal infection may, depending on the phase of gestation, result in abortion, stillbirth, congenital abnormalities, or, when the infection occurs during the first trimester, the birth of immunotolerant, persistently infected, viremic calves [8,9,10]. Persistently infected (PI) calves shed enormous amounts of BVDV throughout their lives, making them a major source for the spread and perpetuation of BVDV within individual cattle herds and transmission to BVDV-free holdings [11,12,13,14]. Therefore, PI calves are the main target of BVD control programs that have been implemented in several countries [13,15,16,17,18]. When PI animals are superinfected with a cp-strain that is antigenically homologous to the persisting ncp-strain or a cp-biotype arises from mutations of the ncp-BVDV already circulating in the animal, the inevitably fatal mucosal disease (MD) develops [7].

In Germany, BVD is a notifiable disease, and a nationwide mandatory eradication program has been in place since 2011. The German control program is primarily based on the testing of every newborn calf within the first month of life for the presence of the BVDV antigen or genome, and the immediate removal of every detected PI animal from the cattle population [15]. Such approaches of testing for virus-positive animals without serological pre-screening have proved beneficial, especially for countries with a high initial BVDV prevalence combined with ongoing vaccination campaigns. This testing approach is highly efficient in reducing the prevalence of persistently infected animals [15], and is without alternatives, since no differentiating infected from vaccinated animals (DIVA) vaccines that would allow vaccination and serological screening are available. 

The German control program has led to a considerable reduction in the prevalence of PI animals over the last nine years. Nationally, approximately 0.5% of calves tested were identified as PI in 2011, and the proportion of PIs amongst newborn calves had decreased to less than 0.01% in 2019. Despite a reduction in the number of PI animals at a national level, differences in PI prevalence exist between individual federal states. In Saxony-Anhalt, the PI prevalence reached 0.0% in 2018 (five PI animals among 156,004 newborn calves in 2016 and no PI animals in 2018, 2019, and 2020) [15,19]. Although voluntary vaccination is allowed in Germany [15], BVDV vaccines are only applied to a limited extent in Saxony-Anhalt. Therefore, there are a large number of naïve and susceptible animals in the national herd, which are at risk of infection if they are confronted with the field virus due to introduction from other federal states or countries.

An alternative approach to BVD testing and monitoring, which has proved beneficial, especially in countries with well-advanced BVD programs, is based on large-scale serology [20]. To screen for the introduction of PI animals into BVD-free herds, bulk milk serology could be used in non-vaccinated herds. Alternatively, spot-testing of young stock older than 6 months of age, to avoid the influence of maternal antibodies, may be used [21,22,23,24,25]. For serological BVD diagnostics, neutralization tests are considered to be the gold standard, since this test system is highly sensitive and allows for differentiation between antibodies induced by BVDV-1 and -2, despite the cross-reactivity that occurs between both BVDV species [26]. Nevertheless, commercially available ELISA systems are used more frequently, as they enable convenient high throughput testing and do not require time-consuming and labor-intensive cell culture systems. However, varying and sometimes unsatisfactory sensitivities were previously described for BVD antibody ELISAs [27,28].

In this study, animals kept in a naturally re-infected herd were blood sampled about seven weeks after the birth of the first PI animal. The collected samples were examined serologically to investigate the capability of commercial ELISA systems for the early detection of a BVDV introduction into a naïve herd. Additionally, we aimed to study the immediate and long term effects of BVDV introduction into a disease-free herd. The study herd was located in the German federal state Saxony-Anhalt and included about 380 cows, their offspring, and fattening bulls at the time of monitoring. The BVDV genome was detected for the first time at the beginning of the year 2017 in two calves during the mandatory ear notch-based testing of every newborn animal. Their mothers were newly introduced into the herd at the end of 2016. Therefore, they were presumably transiently infected in their herd of origin during early gestation, and as a consequence, were pregnant with a PI fetus (so-called “Trojan” cows).

## 2. Results

### 2.1. BVDV Dynamics and Clinical Impact of Virus Introduction into a Naïve Cattle Herd

In January and February 2017 (5 January 2017 and 13 February 2017), two newly purchased cows gave birth to PI calves, which were culled 29 and nine days after birth, respectively, according to the German BVD regulation. Infectious virus could be isolated from their blood leukocytes, and the sequences generated from this sample material belonged to subtype BVDV-1d. Beginning in January 2017, the farmer and farm veterinarian reported a generalized increase in respiratory clinical signs in the herd. However, BVDV could not be definitely confirmed as the causative agent, since blood samples of the in-contact animals were only taken after the second (confirmatory) BVDV-positive test result in a newborn calf. 

**Figure 1:**
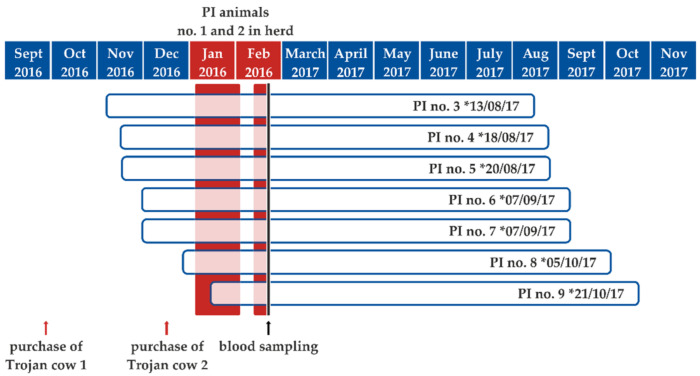
Illustration of the estimated gestation time (blue-framed white bars) for each of the seven persistently infected (PI) calves born in the study herd subsequent to the introduction of the virus into the herd by PI animals no. 1 and 2. The calving dates are indicated at the end of the gestation periods and the phase during which the initial PI calves were present in the herd are shaded in red. The time point of blood sampling is indicated by a vertical black line. * calving date.

On 29 January 2017, a seven-year-old cow showing clinical signs of pneumonia died. In order to ascertain the cause of death, the carcass was sent to the State Institute of Consumer Protection of Saxony-Anhalt for pathological examination. Necropsy revealed a moderate fibrinous to necrotizing bronchopneumonia with intralesional detection of bacteria. *Mannheimia haemolytica* could be isolated from lung lesions. In addition, a moderate hyperplasia of type II pneumocytes was also evident in the lung. The abomasum was filled with greenish-brown fluid and an acute erosive and ulcerative abomasitis was evident. The small intestine also contained fluid ingesta. The caecum and colon showed no abnormal content. Unfortunately, autolysis impaired the histological analysis of the intestinal mucosa. The remaining organs were macroscopically inconspicuous. By conducting real-time PCR analysis, BVDV and *Mycoplasma bovis* were detected in a tissue pool sample including nasal mucosa, lung, lymph nodes, abomasal and intestinal mucosa, and brain tissue. Infections with bovine herpes virus type 1 (BoHV-1), bovine parainfluenza virus 3 (BPI-3), bovine respiratory syncytial virus (BRSV), and *Chlamydia* sp. could be excluded. However, infectious BVDV could be isolated from the tissue pool in cell culture. This virus isolate of a cow that succumbed 24 days after the birth of the first PI calf belonged to subtype BVDV-1d. The 5′UTR sequence generated was 100% identical to those from the first two PI calves, indicating that the PI calves represented the source of infection. The serological follow-up of the group kept in the same stable department (stable group; please see below) showed that nine of the 18 animals in contact with the Trojan cows seroconverted within 48 days after the birth of the first PI calf. Four of these seropositive animals, as well as three further dams, which did not belong to the primarily affected stable group, gave birth to PI calves themselves in the following months. The calving dates ranged from 13 August 2017 to 21 October 2017 (Figure 1). The estimated gestation time of the seven newly infected cows was calculated by subtracting the mean cattle gestation period of 283 days from the calving date. The gestation time ranged between days 10 and 63 of pregnancy on the day of the birth of the first PI calf from the purchased Trojan cows and between days 42 and 111 of pregnancy on the day when the second initial PI animal was removed from the herd. This presumed infection period is shown in Figure 1.

To summarize the virus dynamics within the described study herd, the birth of PI calves from the newly purchased Trojan cows led to acute BVDV infections, as confirmed by virus detection in a succumbed cow, while a generalized increase in respiratory infections in the herd was reported. Moreover, several further PI calves were induced by infections of their mothers during pregnancy. The birth dates started roughly half a year after the birth of the first PI calf, and all calves had to be culled according to the German BVD regulation. Therefore, BVDV had a significant impact on the study herd, even several months after virus introduction. 

Following the births of these PI calves and their removal, no further BVDV cases have been diagnosed since 2017. Consequently, the outbreak could be efficiently stopped by the immediate culling of all newborn PI animals, the applied vaccination, and adhering to strict biosecurity measures.

### 2.2. Serological Investigation of the In-Contact Dams

Forty-eight days after the birth of the first PI calf, a serum sample was taken from each of the 18 cows that were in close contact with the Trojan cows during the parturition period (stable group). The sera were investigated by a microneutralization test and eight different commercially available antibody ELISAs. In nine of the 18 in-contact animals (50.0%), anti-BVDV antibodies were detected by the microneutralization test. However, when analyzed by different commercially available BVD antibody ELISAs, a high variation of the seropositivity rate was observed. While ELISA A (ID Screen BVDV p80 Ab competition) showed 100% accordance with the neutralization test, four of the other applied ELISAs only exhibited 83.3% conformity (Table 1). By using ELISA D (Svanovir BVDV Ab Screening), ELISA E (Svanovir BVDV Ab biphasisch), and ELISA G (Prio-CHECK^®^ BVDV Ab), three sera scored negatively, although they had reacted positively in the neutralization test (titers between 1/11 and 1/18). ELISA B (IDEXX BVDV Ab total) did not identify two sera as being positive, and the neutralizing titers were 1/14 and 1/18. In addition, one negative sample resulted in a doubtful measuring range of this ELISA. Only 72.2% accordance compared to the neutralization test was obtained by ELISA C (IDEXX BVDV p80Ab), which did not detect five out of nine sera with neutralizing antibodies (titers between 1/11 and 1/143). When employing ELISAs F (Svanovir BVDV p80 AB) and H (Serelisa BVD/MD AB Mono Blocking), only 61.1% of the sera were interpreted correctly (Table 1). While ELISA F did not detect seven out of nine antibody-positive sera (neutralizing titers between 1/11 and 1/143), four sera produced false-negative results in ELISA H (titers between 1/11 and 1/18), and some of the SNT-negative samples were false-positives or doubtful. 

In conclusion, the seroprevalence of the stable group ranged from 11.1% to 50.0%, depending on the serological test system applied (Table 1).

## 3. Discussion

Due to the virus’ considerable impact on animal health and welfare, numerous countries have implemented campaigns to eradicate BVDV from their cattle populations, which have been proven to be highly effective and successful [13,15,18]. However, especially during the final phase of such control programs, a large number of animals are left naïve and susceptible to infection if confronted with the virus, which may still be present in the cattle population at a very low level, or which may be brought in from other countries. Therefore, if preventative biosecurity measures are insufficiently imposed, or the awareness of farmers and stakeholders declines over time, then BVD-free holdings or regions are at risk of reinfection. 

In the context of BVD, Trojan cows are a major cause, in addition to PI animals, for BVDV spread into hitherto unaffected holdings [11,12,13,14,29] and represent a particular challenge when controlling BVDV. In such cases, the problem only becomes visible after the birth of the PI calf, which might be several months after the purchase of the pregnant dam. For the early detection of BVDV infections during pregnancy and to identify dams at risk for the delivery of a PI calf, serological methods might be beneficial, provided they are applied at regular intervals. Furthermore, routine serological examinations could be used on a herd level to monitor for virus introduction. Although the detection of neutralizing antibodies by the microneutralization test is considered the gold standard, ELISA systems are used much more frequently due to the combination of easy and convenient handling and the facilitation of rapid high-throughput testing. However, in this study some of the applied ELISAs tested single serum samples incorrectly negative. This observation that varying and sometimes unsatisfactory sensitivities can occur was also made in previous comparisons of BVD antibody ELISAs. Hence, thorough validation and careful selection of ELISA tests are necessary. Some BVD ELISAs have been described to be less sensitive for antibodies induced by immunization with inactivated vaccine preparations [27,30,31,32]. However, in this study, we only tested animals that were naturally infected by contact with a PI calf. Further reasons for varying sensitivities could include the ELISA production process itself or the antigen used for plate coating, but in this study, differing results were also produced with ELISAs that rely on the identical viral protein. Here, only one commercial test correctly detected all animals in which neutralizing anti-BVDV antibodies were detectable, while all other tests underestimated the seroprevalence, in some cases dramatically. 

Even more important than the misjudged herd seroprevalence is the fact that not every newly infected pregnant dam was identified by each of the applied tests, potentially leading to overlooking a Trojan cow, although such cows pose a significant risk for BVDV spread [11,12,13,14,29]. Once the PI calf is born, it is highly efficient in spreading the virus and, when naïve dams are infected, in inducing further PI animals, as has been once again demonstrated in this study. Moreover, the negative effects of BVDV introduction into a hitherto negative cattle herd may persist for several months. The critical period for the induction of persistent BVDV infections is the first trimester of the 9-month gestation up to about day 150 of gestation [10,33]. As the new PI calves were born at term in the study herd, there was roughly half a year between the infection of the dams and the births of their BVDV-infected offspring. This time period illustrates the serious, long-running impact of BVDV introduction into a herd and demonstrates the protracted nature of an eradication process, if implemented. To prevent further transmission of the virus and the establishment of transmission chains in cattle herds, potential Trojan cows and in particular their offspring should be separated from susceptible animals until a negative virological test result has been obtained for the calf or, in the case of positive test results, until the newborn PI calf is removed from the herd. However, to impose this precautionary measure, the dam needs to be identified as being at risk. 

Considering the role of Trojan cows in the spread of BVDV, this important animal group came into focus of the control program when the German BVD regulation was adjusted in June 2016. Among others, movement restrictions were prescribed for farms with PI animals, consisting of a period of 40 days for every unvaccinated cattle and for pregnant dams until birth to stop the movement of possible Trojan cows to unaffected holdings. 

On an international level, the importance of import investigations and the role of Trojan cows is, for example, also reflected in the new Animal Health Law (AHL), which will come into force in the European Union in April 2021. According to the new regulations, pregnant animals that are transported from not yet disease-free regions to BVD-free holdings have to be tested negative for antibodies against BVD after a 21-day quarantine or, alternatively, positive for antibodies against BVD before conception. However, for animals transported from BVD-free regions, no further investigations are required according to the AHL. Monitoring for the maintenance of the status will be mainly based on serological investigations conducted once a year. Therefore, a reintroduction of BVDV in free herds might only be discovered after several months, and in the worst case, even several weeks after the birth of the PI animal. Hence, when forgoing to test pregnant cows for the presence of BVD antibodies prior to transport between free regions, the risk of re-housing undetected Trojan cows might be very low, but it still exists, especially when using antibody test systems with a low sensitivity for monitoring. Therefore, both the test intervals and the test system need to be selected very carefully. As shown in our study, seroprevalences in herds and the correct identification of antibody-positive individuals vary markedly, depending on the applied ELISA test. Therefore, a thorough quality check for antibody ELISAs used for this purpose is strongly recommended. Nevertheless, despite the imponderables, the new AHL regulations allow, for the first time, additional guarantees for countries which are officially free of BVDV or which have an accepted control program. As implemented for BoHV-1 for many years, the new EU regulations can help maintain a hard-won status. However, the new rules also have weaknesses, such as a lack of uniform test rules, the consequences of which must be considered in the future.

All cows kept in the study herd were vaccinated against BVDV after virus introduction became evident, as vaccination represents an additional approach of disease prevention [13]. However, as the stable group was only vaccinated several weeks after the birth of the first PI calf and therefore after infection of the naïve pregnant dams, the induction of further PI animals could not be completely prevented. Nevertheless, vaccination might have contributed to disease eradication from the cattle herd, as the PI animals born from August to October 2017 did not induce further PI animals, and no BVD case occurred in the years 2018 to 2020 [19]. 

When using vaccination as an additional tool in disease eradication approaches, some crucial factors should be kept in mind. At least all susceptible breeding animals have to be vaccinated in the herd, with the vaccine being used correctly according to the manufacturer’s instructions [34,35]. However, a major disadvantage of vaccinating naïve herds includes the loss of the possibility to use milk serology to screen for virus introduction, as antibodies induced by vaccination cannot be differentiated from naturally-derived antibodies subsequent to infection. Nevertheless, after virus introduction, the same restrictions regarding serological monitoring also apply due to the induction of antibodies by the field infection. Hence, in such herds, vaccination might represent a useful additional tool for disease control. 

While a major animal health and welfare impact of BVDV originates from the birth of PI calves that often succumb to the virus’ late-onset form of the inevitably fatal MD, BVDV is also described to be a key pathogen in the multifactorial bovine respiratory disease (BRD) complex [36,37,38]. Therefore, the clinical signs indicative of a respiratory disease that were observed during the period in which the PI calves were present in the study herd might be related to BVDV. However, BVDV could not be confirmed by laboratory diagnostics as the causative agent, since samples of the stable group were only taken after the second (confirmatory) BVDV-positive test result in a newborn calf. Nevertheless, the virus was isolated from a succumbed cow, and the 5′UTR sequence of this virus was identical to that of the PI animals. Therefore, it can be assumed that the PI calves born by the purchased Trojan cows infected an adult cow and led, due to a presumed BVD-induced immunosuppression [7,9], together with *Mannheimia haemolytica* and *Mycoplasma bovis*, to multifactorial pneumonia with a fatal outcome. This observation of a lethal course of disease is in line with previous descriptions of BVDV re-introductions into disease-free herds, which led to serious illness up to the death of animals not only in young calves, but also in adult cows [12,17,39]. 

To conclude, BVDV introduction had a strong, long-lasting negative impact on the hitherto disease-free study herd, due to a generalized increase in respiratory infections and the loss of animals, be it by the disease itself (i.e., the succumbed adult cow) or by culling of the newborns according to the national BVD regulation. Therefore, purchase investigations should include control of the BVD status, when animals are re-housed from not yet disease-free holdings or regions to free areas. 

## 4. Materials and Methods

### 4.1. Cattle Holding and BVDV Introduction into the Study Herd 

A BVD virus-free private dairy cattle holding located in the German federal state Saxony-Anhalt was monitored. At the time of the study, around 380 cows (beef cattle) and their offspring, as well as fattening bulls, were kept there. Heifers and cows were housed in several stable groups. Vaccination against BVD was not routinely implemented before the study period, i.e., prior to the birth of the first PI calf. 

The BVDV genome was detected in a newborn calf at the beginning of the year 2017. Epidemiological investigations revealed that its mother and a further cow, which also gave birth to a PI calf several days later, were introduced into the study herd in September and December 2016 (Figure 1). Both newborn animals tested positive for the BVDV genome during the ear notch-based mandatory testing of every newborn calf in the holding. For confirmation of the BVDV test results, blood samples were collected from the newborn calves according to the German BVD regulation and likewise tested positive for the BVDV genome. The PI calves were culled immediately after the confirmatory BVDV positive RT-PCR results 29 and nine days after birth, respectively. 

To limit the negative effects of the BVDV introduction on pregnant dams, all cows were vaccinated with an attenuated live vaccine against BVD (Bovela©, Boehringer Ingelheim Vetmedica GmbH, Ingelheim/Rhein, Germany) 48 days after the birth of the first PI calf. Prior to vaccination, a serum sample was taken from each of the 18 cows that were in contact with the Trojan cows (stable group).

### 4.2. Pathological, Bacteriological, and Virological Investigation

A 7-year-old cow that died in January 2017 was necropsied, and the following samples were taken for molecular and bacteriological analyses: Lung; tracheobronchial lymph nodes; trachea; nasal mucosa; brain tissue; abomasal and intestinal mucosa including Peyers`s patches; and intestinal lymph nodes. For further investigation, the tissue samples were homogenized (PrioGENIZER, Thermo Fisher Scientific GmbH, Darmstadt, Germany) and nucleic acid was extracted using the QIAmp cador Pathogen Mini Kit (Qiagen GmbH, Hilden, Germany), according to the manufacturer’s instructions. 

The ear notch samples taken routinely from every newborn calf were incubated for 45 min at 70 °C in 450 µL of an in-house direct lysis buffer consisting of 4.6% proteinase K (20 mg/mL, Qiagen, Hilden, Germany), 44.4% RLT buffer (Qiagen, Hilden, Germany), 1.5% carrier RNA (Qiagen, Hilden, Germany), 1.2% DL-Dithiothreitol (Sigma-Aldrich, Taufkirchen, Germany), and 48.3% RNAse-free water. The lysis reaction was followed by the purification of total RNA using the RNeasy Mini Kit (Qiagen GmbH, Hilden, Germany), according to the manufacturer’s instructions. 

RNA extracted from organ and ear notch samples was subsequently tested for the BVDV genome by real-time RT-PCR using the LSI Vetmax^TM^ BVD4all Real-time RT-PCR kit (Life Technologies, Darmstadt, Germany). Nucleic acid extracted from the organs of the succumbed cow was additionally tested by real-time (RT-)PCR for BoHV-1 [40], BPI-3 [41], BRSV [42], and *Chlamydia* sp. [43].

Virus isolation was carried out on KLu-2-R cells (CCLV-RIE 091, Collection of Cell Lines in Veterinary Medicine, Insel Riems, Germany) at 37 °C in a 2.5% CO_2_ atmosphere for five to seven days. For the inoculation of cells, the leukocyte fractions from blood samples of the PI calves were isolated by centrifugation (1500× *g* for 10 min), followed by the specific lysis of erythrocytes (Erythozyten-Lysepuffer; CC-pro, Oberdorla, Germany). Organ samples of the succumbed cow were homogenized as described above and after one freeze/thaw cycle, the material was centrifuged (2000× *g*, 10 min), followed by sterilizing filtration. The resulting material was used for the inoculation of cells. Total RNA from tissue culture supernatant was extracted using the QIAamp Viral RNA Mini Kit (Qiagen GmbH, Hilden, Germany) and the presence of the BVDV genome was confirmed by real-time RT-PCR (LSI Vetmax^TM^ BVD4all Real-time RT-PCR, Life Technologies). For molecular typing of the virus, the 5′ UTR genomic region was sequenced as described previously [44].

For the detection of bacterial pathogens, the organ samples of the succumbed cow were plated onto blood agar dishes (Meat Extract Peptone Sheep Blood Agar 5%, OXOID, Heidelberg, Germany) and incubated aerobically at 37 °C for 24 to 48 h. Pure colonies of a single colony style were further analyzed using the matrix-assisted laser desorption/ionization time-of-flight mass spectrometry method (MALDI-TOF MS [45]). 

### 4.3. Serology

The sera taken from the in-contact cows prior to vaccination were analyzed by a microneutralization test against the heterologous BVDV-1a strain NADL, as described in the German official collection of test methods for bovine viral diarrhea [46]. In addition, the sera were tested by the following commercially available antibody ELISAs, according to the manufacturer’s instructions: (A) ID Screen BVDV p80 Ab competition (ID.vet, Grabels, France); (B) BVDV Ab total (IDEXX, Liebefeld, Switzerland); (C) BVDV p80AB (IDEXX, Liebefeld, Switzerland); (D) Svanovir BVDV Ab Screening (Svanova, Uppsala, Sweden); (E) Svanovir BVDV Ab biphasisch (Svanova, Uppsala, Sweden); (F) Svanovir BVDV p80 AB (Svanova, Uppsala, Sweden); (G) PrioCHECK^®^ BVDV Ab (Thermo Fisher Scientific, Waltham, Massachusetts); and (H) Serelisa BVD/MD AB Mono Blocking (Zoetis, Berlin, Germany). Results were interpreted according to the manufacturer’s instructions for the respective ELISA. Doubtful results were considered positive.

## Figures and Tables

**Table 1:** Serological analyses of animals that were in close contact with the newly purchased Trojan cows during their parturition period. Positive or doubtful results are presented in bold, and the results of commercial BVDV antibody ELISAs that did not agree with the results of the microneutralization test are shaded in gray (doubtful results were considered positive).

Sample Number	Neutralizing Titer	Commercial ELISA Kit							
		A	B	C	D	E	F	G	H
165285100-36	**1/113**	**positive**	**positive**	**positive**	**positive**	**positive**	**positive**	**positive**	**positive**
165291382-46	**1/14**	**positive**	negative	negative	negative	negative	negative	negative	negative
165285930-52	**1/14**	**positive**	**doubtful**	negative	**positive**	negative	negative	**positive**	negative
165286867-59	**1/143**	**positive**	**positive**	negative	**positive**	**positive**	negative	**positive**	**positive**
165284904-62	**1/18**	**positive**	negative	negative	negative	negative	negative	negative	negative
165284894-64	**1/227**	**positive**	**positive**	**positive**	**positive**	**positive**	**positive**	**positive**	**positive**
165285390-97	**1/57**	**positive**	**positive**	**doubtful**	**positive**	**positive**	negative	**positive**	**doubtful**
165284012-98	<1/5	negative	negative	negative	negative	negative	negative	negative	negative
165287229-114	<1/5	negative	negative	negative	negative	negative	negative	negative	negative
165286100-115	<1/5	negative	negative	negative	negative	negative	negative	negative	negative
165285846-116	<1/5	negative	negative	negative	negative	negative	negative	negative	negative
165283770-117	<1/5	negative	negative	negative	negative	negative	negative	negative	negative
165286969-120	<1/5	negative	negative	negative	negative	negative	negative	negative	**positive**
165284481-123	<1/5	negative	negative	negative	negative	negative	negative	negative	**doubtful**
165285579-137	<1/5	negative	negative	negative	negative	negative	negative	negative	**doubtful**
165286082-142	**1/22**	**positive**	**positive**	**doubtful**	**positive**	**positive**	negative	**positive**	**doubtful**
165285832-147	**1/11**	**positive**	**positive**	negative	negative	**positive**	negative	negative	negative
165284512-163	<1/5	negative	doubtful	negative	negative	negative	negative	negative	negative
∑ positive or doubtful	9/18 (50.0%)	9/18 (50.0%)	8/18 (44.4%)	4/18 (22.2%)	6/18 (33.3%)	6/18 (33.3%)	2/18 (11.1%)	6/18 (33.3%)	8/18 (44.4%)

## Data Availability

The data obtained in this study are available in the article.
